# Well-Being in the Context of COVID-19 and Quality of Life in Czechia

**DOI:** 10.3390/ijerph19127164

**Published:** 2022-06-10

**Authors:** Patrik Maturkanič, Ivana Tomanová Čergeťová, Irena Konečná, Vladimír Thurzo, Amantius Akimjak, Ľubomír Hlad, Jan Zimny, Marie Roubalová, Victoria Kurilenko, Martin Toman, Jozef Petrikovič, Lucia Petrikovičová

**Affiliations:** 1 Faculty of Roman Catholic Theology of Cyril and Methodius, Comenius University Bratislava, 81458 Bratislava, Slovakia; patrikmat@seznam.cz (P.M.); vladimir.thurzo@frcth.uniba.sk (V.T.); 2 College of Applied Psychology, 41155 Terezín, Czech Republic; cergetova.ivana@gmail.com (I.T.Č.); konecna@vsaps.cz (I.K.); 3 Department of Social Works, Faculty of Theology, Catholic University in Ružomberok, 03401 Ruzomberok, Slovakia; amantius.akimjak@gmail.com; 4 Department of Religious Studies, Faculty of Arts, Constantine the Philosopher University in Nitra, 94901 Nitra, Slovakia; lhlad@ukf.sk; 5 Akademia Wojsk Lądowych Imienia Generała Tadeusza Kościuszki we Wrocławiu, General Tadeusz Kościuszko Military University of Land Forces in Wrocław, Czajkowskiego 106, 51-147 Wrocław, Poland; jzimny@przemysl.opoka.org.pl; 6 Department of Biblical and Jewish Studies, Hussite Theological Faculty, Charles University in Prague, 14000 Prague, Czech Republic; marie.roubalova@htf.cuni.cz; 7 Russian Language Department, Medical Institute, Peoples’ Friendship University of Russia, 117198 Moscow, Russia; vbkurilenko@gmail.com; 8 Martino—Institute of Society Development, 82104 Bratislava, Slovakia; martin@myjump.sk; 9 Department of Ecology and Environmental Sciences, Faculty of Natural Sciences and Informatics, Constantine the Philosopher University in Nitra, 94901 Nitra, Slovakia; jozef.petrikovic@ukf.sk; 10 Department of Geography, Geoinformatics and Regional Development, Faculty of Natural Sciences and Informatics, Constantine the Philosopher University in Nitra, 94901 Nitra, Slovakia

**Keywords:** well-being, pastoral and psychological service, pandemic

## Abstract

The present study focuses on exploring the differences and relationship between well-being and experience of pastoral and psychological service of religious denomination based on religious affiliation during the first wave of the pandemic in Czechia. Our research has been focused on the investigation, comparison, and correlation between the level of well-being and pastoral and psychological service. The research sample (*n* = 1126) consisted of the Czech health population with age over 16 years, of which 42.4% were men (*n* = 478) and 57.5% were women (*n* = 648). From the perspective of religiosity, the study sample was divided in terms of religion into two groups—51.9% participants with religious affiliation (*n* = 584) and 48.1% participants without religious affiliation (*n* = 542). The level of well-being was identified by means of The Satisfaction with Life Scale (Diener, Emmons, Larsen, & Griffin, 1985). The level of experience with pastoral and psychological service was measured using our non-standardised questionnaire. The results confirmed the differences between the variables of well-being and positive experience with pastoral and psychological service based on religious affiliation. Moreover, we confirmed the hypothesis of a positive correlation between well-being and positive experience with pastoral and psychological service in Czechia.

## 1. Introduction

**Figure 1:**
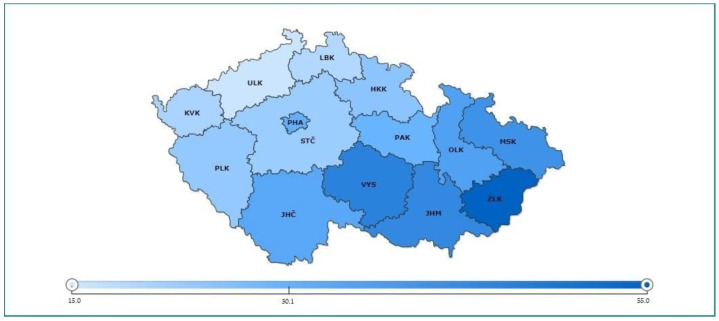
Share of believers in %.

In 2020, the disease COVID-19 appeared in the Czechia and worldwide, resulting in a crisis due to the pandemic. Restrictive measures have cancelled most organisations, and their activities have been suspended or terminated. The helping professions, which focused on pastoral and psychological service [1,2]—which is characterised by pastoral counselling, care and psychotherapy refer to the structures adopted by the clergy to assist their members and other clients promote personal and social development in the religious and spiritual realm—were found themselves in a position of social exclusion [3], religious intolerance [4,5] and also unable to carry out their activities and were forced to look for alternative ways of supporting the sick as well as the grieving people and families. Religious intolerance was associated mainly with special exceptions related to the government’s restrictive measures in Czechia. It is crucial to say that the vast majority of the Czechia is an atheistic and agnostic country. Based on the 2021 census, 18.7% of those who filled the question declared a religious faith to be a believer and to belong to a church or religious society. The answer without religious faith accounted for more than two-thirds (68.3%) of the answers. Completion of the question on religious faith was voluntary, 30.1% of people left it blank, while in the 2011 census it was 44.7% (Figure 1) [6]. Based on these data and following our practice, we decided to realise research focused on investigating, comparing, and correlating the level of well-being and subjective experience of pastoral and psychological service during the first wave of the pandemic in Czechia.

The study of well-being in various situational contexts is currently emerging with a topic closely related to the pandemic situation and the quality of life of a person in his current living conditions [7,8]. Many local and international studies focus on the issue of organisations, which were paralysed in their activities such as work, education [9,10,11,12], university studies [13], or community. Scientists from many disciplines have begun to describe the 2020 pandemic connected with COVID-19 in various scientific contexts, seeking to find factors related to the crisis [14]. In general, quality of life has started to be discussed intensively from various scientific perspectives.

The aim of the paper is to research and compare the level of well-being and subjective experience of pastoral and psychological service during the first wave of the pandemic in the Czechia. The research focused on quality of life is an interdisciplinary issue among the humanities and brings significant findings indicating intonational documents affect the country’s global sustainable development [15,16,17,18,19,20,21]. However, due to applying the problem in practice, many scientific disciplines, including medicine, sociology, economics, theology, political science, and psychology, deal with their future lives. The data obtained based on extensive findings are mostly of official significance. The World Health Organisation (WHO) medical perspective defines the quality of life as the perception of one’s position in culture, values, expectations, and norms of society (free translation www.who.int, accessed on 22 April 2022). From the health point of view, the quality of life is focused mainly on health and related problems. The socio-economic and socio-cultural quality of life [22,23] points to the population’s standard of living and society as a global macro group, primarily based on geographical location as a nation, or regional population, with the most frequently identified household being the smallest functional unit.

The psychological concept of quality of life is based on subjective satisfaction and well-being, or dissatisfaction and discomfort of individuals with their own lives. The pastoral concept of quality of life is very similar [24,25,26,27], and we can perceive a connection with the themes of ethics and morality [28,29,30,31]. Compared to the socio-economic point of view, the functional unit does not become the household but the individual and his perception of personal experience. The topic of quality of life is dealt with in the psychological scientific field by representatives of positive psychology, who consider its building to be the goal of this direction [32]. From a psychoanalytic point of view, we look at the quality of life as the ability to love, work, and live according to a given culture [33]. According to behavioural psychology, we can define quality life as a set of effective habits and skills that lead to permanent health and well-being [34]. Among several authors, Diener’s definition of well-being is important [35], which defines it as (1) an individual’s emotional response to life events, (2) satisfaction, and (3) overall cognitive evaluation of satisfaction with life. Compared to the previous author, [36] extends the concept of subjective well-being to six dimensions (autonomy, control of the environment, personal growth, positive relationships with others, the meaning of life and self-acceptance). From the point of view of Keyes and Lopez [37], well-being comprises social acceptance, updating, contribution, cohesion, and integration.

The concept of well-being came in 1984 when the WHO identified it as one of the crucial parts of health. In general, it is a term that fits into the concept of positive psychology, which is a purely psychological concept [38]. Well-being is formed by emotional and cognitive dimensions—evaluating one’s own life [39], such as positive and negative affectivity, happiness, life satisfaction, or moods [40]. Ref. [35] perceive well-being as a broad category of phenomena, including “an individual’s emotional responses, satisfaction in the domains of life, and a global assessment of life satisfaction” (p. 277), based on data from the interviewee’s own experience [41]. In the search for critical factors supporting well-being, we decided to rely on [36], who defined six basic dimensions closely related to the self-determination theory. If we try to name these factors, we are talking about the following areas: (a) Self-acceptance as a positive attitude towards oneself, knowledge of one’s own emotions and cognitions, acceptance of one’s own good and wrong sides and coping with one’s own past; (b) personal development as an experienced feeling of constant growth and development, openness to new experiences, the need to realise one’s own potential and the ability to see change for the better in one’s own behaviour; (c) the meaning of life as the feeling of meaningfulness of the present and past life, the ability to set oneself fulfilling life goals, to think about the meaning and purpose of life; (d) managing the environment as experiencing a sense of competence and manageability of one’s environment, an overview of what is happening in the environment, effective use of opportunities, the ability to choose or create an environment suitable for meeting one’s own needs and achieving values; (e) autonomy as the independence and self-determination, the ability to resist social pressures and maintain one’s own way of thinking and acting, independence from judgment by others; (f) positive relationships with people as warm, satisfactory and confidential relationships with other people, interest in the well-being of others, the ability to empathise, intimacy and reciprocity in relationships.

In connection with the pandemic situation, we encountered a significant reduction in the quality of life and its survival. During the first wave of the pandemic, many restrictive measures were imposed that curtailed fundamental human rights and caused human suffering [42]. The key to psychological pressure was social isolation, free movement restriction, increased pressure for individual performance, exclusion from social groups (family, work, or community) and reduced spiritual activities [43]. The religious denomination was severely limited in activities and did not have the opportunity to contribute to individuals’ lives actively. Several situations have emerged that emphasise human invulnerability as loneliness, abandonment, helplessness or irrational beliefs [44], which have also been associated with increased mortality of loved ones and acquaintances during a pandemic. The religious denomination’s role was reduced, and people had to be satisfied with individual communication with God as an attachment person [45], which the religious denomination could not fully represent at the time. The emotional responses, which also occur in humans during a pandemic, can be divided into two relatively separate dimensions: positive affectivity and negative affectivity. These are practically a living assessment of the events around the individual in which he is currently located. The global evaluation of life satisfaction represents the cognitive appreciation of an individual’s life as a whole [46,47,48] point to assessing satisfaction with life as general or only with particular aspects of life. One of these aspects is the spiritual environment and religion [49] confirm that social relationships are a very significant segment of well-being and are related to the subjective evaluation of the individual, which he judges on the experience experienced with the degree of well-being. According to the authors’ research, people show a higher level of well-being when they experience emotionally stronger partnerships and social relationships, are more inclined to marry in personal relationships, and better manage conflicts.

## 2. Materials and Methods

Our research has focused on investigating, comparing and correlating the level of well-being and subjective experience of pastoral and psychological service during the first wave of the pandemic in Czechia. The following research questions are addressed in the study: Are the variables well-being and quality of experience with pastoral and psychological service based on religious or nonreligious affiliation different? Second, are there relationships between the variables of well-being and quality of experience with pastoral and psychological service? The present study aims to test whether there is a positive relationship between religious affiliation and well-being. We assume that the quality of experience with pastoral and psychological service will decrease with the religious affiliation.

A total of 1126 respondents participated in the research: 42.4% men (*n* = 478) and 57.5% women (*n* = 648)—age divided into six groups: under the age of 18 years (3.8% of participants, *n* = 43), from 18 to 25 (9.2% of participants, *n* = 104), from 26 to 40 (25.7% of participants, *n* = 289), from 41 to 59 (44.8% of participants, *n* = 505), from 60 to 89 (15.1% of participants, *n* = 170) and over 90 (1.3% of participants, *n* = 15). The research group consisted of the Czech population aged 16 and over. The research participants had different levels of education: 5.9% with basic education (*n* = 66), 41.5% with secondary education (*n* = 467) and 52.7% with higher education (*n* = 593). From perspective of religiosity, the study sample was divided in terms of religion into two groups—51.9% participants with religious affiliation (*n* = 584) and 48.1% participants without religious affiliation (*n* = 542).

The research included a general sociodemographic questionnaire and basic religion identification part, followed using another test standardised method and questions about experience with pastoral and psychological service in pandemic situation COVID-19 during the first wave of the disease. We used two questionnaires to measure well-being and describe experiences with pastoral and psychological service during the research.

The well-being level has been identified with The Satisfaction with Life Scale (SWLS) [50]. This scale is a unifactorial self-report questionnaire consisting of five items (e.g., “I am satisfied with my life.”). The SWLS uses five response alternatives (five-point on a Likert scale—from (1) totally disagree to (5) totally agree). This scale is narrowly focused on assessing global life satisfaction and correlated moderately to highly with other measures of subjective well-being. SWLS has adequate psychometric properties in our sample, with reliability of α = 0.85. When evaluating the results, we worked with the total score achieved. The second questionnaire was non-standardised and created from 10 questions (five-point on a Likert scale), which focused on personal experience with COVID-19 disease and pastoral and psychological service (PPSE) of the religious denomination during the first wave of the pandemic situation (e.g., “I noticed that the religious denomination was significantly involved in helping the grieving citizens during the pandemic.”). We also asked several questions that were the subject of descriptive analysis and qualitative analysis of the answers.

The data collection phase was conducted from April to July of 2021 in the COVID-19 pandemic situation, during the first wave of the disease. Data were obtained online from research participants using a “snowball technique” for data collection. We contacted people personally and via e-mail who met the criteria of our research—adults living in the Czechia. All participants of the research agreed with the conditions of research and have been informed about consent complying with ethical and research standards. The participants’ right to anonymity and the confidentiality of their results were guaranteed. The response rate was 74%. No incentives were provided to participate in the research. Recruitment of participants was carried out in the form of direct contact. Before participants started to respond to the online questionnaire, they were given all the necessary information about the study and instructions on how to respond. The online questionnaire was administered individually without a time limit. All research participants agreed to the terms of the study and were informed of the agreement following ethical and research standards. Data were collected anonymously and evaluated using a statistical program SPPS (Version 23 for Windows, IBM, Armonk, NY, USA). Descriptive statistics were used to give an indicator of mean scores on subscales of well-being and type of religiosity in the healthy Czech population (general sample). The study used the correlation research design, whereby we analysed the frequency of particular items and correlations by means of correlation analysis between cardinal variables using the Pearson coefficient representing the linear dependence between the two variables, which is used in the normal distribution of data. The tightness of the relationship was assessed for 5% and 1% of the level of statistical significance.

## 3. Results

The first step was to obtain the values achieved by the Czech healthy population as the average, standard deviation, minimum and maximum score values and then dividing into two groups based on religious and nonreligious affiliation in the questionnaire SWLS [50,51], as presented in Table 1. Table 2 presents the differences between the well-being and positive experience of pastoral and psychological service (Mann–Whitney non-parametric scale). There is a significantly higher score in variables of SWLS and PPSE in participants with religious affiliation (Table 1). Furthermore, there is a significant difference in both variables between these two groups, as seen in Table 2. We also represented the finding of correlation analysis. The results confirmed the relationship between well-being and positive experience with pastoral and psychological service in a positive direction (r = 0.350) at the level of significance *p* < 0.01.

The part of the qualitative analysis gave us some basic information about participants’ experience with the pandemic and the pastoral and psychological service of the religious denomination. The first answers were connected to the question, “Do you have someone around you who died of covid-19?”. There was no direct experience with COVID-19 disease and death in 42.8% of participants (*n* = 482). Participants identified death from COVID-19 in their area in 9% of distant relatives (*n* = 101), 33.4% of friends or acquaintances (*n* = 376) and 8.5% of close relatives (*n* = 96). 6.3% of participants (*n* = 71) refused to answer this question. 

In our next question, we asked: “Did you have the opportunity to “accompany” (a form of farewell) your loved one or acquaintance dying with COVID-19 at the moment of his departure for the Second World?” Unfortunately, 82.5% of research participants (*n* = 989) did not have this opportunity.

We also asked: “Did you attend the last farewell, the funeral of the person who died from COVID-19?”. Unfortunately, a farewell or funeral of a dead person at COVID-19 was attended by only 14.6% of participants (*n* = 163). 

During the first wave of the pandemic situation, the religious denomination carried out a nationwide call for the “ringing of bells” in memory of the first victims of the Czech covid and post-COVID-19 deaths (22 March 2021). We asked our respondents if they had heard of this special call realised by the religious denomination. Of the research participants, 33.3% did not hear about the call (*n* = 375). On the other hand, 35.4% of respondents noticed this call passively (*n* = 398), and only 31.3% of research participants actively participated (*n* = 352).

We also asked whether the participants noticed that the religious denomination would have been involved in helping the grieving citizens in pastoral and psychological service during the pandemic events. 12.3% of research participants answered positively (*n* = 139), 22.5% of people noticed this marginally (*n* = 253). A total of 65.2% (*n* = 734) of respondents did not notice any activity of the religious denomination in pastoral or psychological service during the pandemic. 

We were interested in whether respondents participated in the religious denomination’s activities during the pandemic, connected with the pastoral and psychological service. Unfortunately, only 23.8% (*n* = 268) of research participants were interested in community activities or services during the pandemic in community contribution and 8% (*n* = 99) in religious denomination customs sense. Under certain circumstances, 32.4% (*n* = 365) of respondents thought about it, but 35.0% (*n* = 394) were not interested in pastoral and psychological service.

In the last question of our qualitative part of the research, we asked whether this global and personal suffering in the loss of loved ones taught you to think more about life and death. 30.6% (*n* = 345) of people said that the pandemic had not changed their lives, and I take life as it goes. On the other hand, 20.2% (*n* = 227) learned to think more about earthly life and 14.9% (*n* = 168) learned to think more about earthly but also the afterlife, and 34.3% (*n* = 386) that it taught them not only to think more about early but also eternal values in the context of life practice.

## 4. Discussion

The present study’s primary purpose was to explore the differences and relationship between well-being and experience of pastoral and psychological service of religious denomination based on religious affiliation during the first wave of the pandemic in Czechia. The results confirmed the differences between the variables of well-being and positive experience of pastoral and psychological service based on religious affiliation. Moreover, we confirmed the hypothesis of a positive correlation between well-being and positive experience with pastoral and psychological service of the religious denomination in Czechia.

Due to the COVID-19 pandemic situation in 2020, we had the opportunity to realise that social interactions are a crucial part of our daily lives. If we are not isolated individuals, we encounter interpersonal interactions constantly throughout our life journey [2]. Man thus develops into an individual, communicative and social-social creature in many environments [52], including psychological and pastoral service [53,54,55,56,57].

During the COVID-19 pandemic, believers experienced a physical and mental balance, which helped them to better overcome not only their own difficulties, but also their immediate surroundings. The research showed that the element of transcendent values was a strong support for men and women who subscribed to religions (questions), where the psychological aspect played an important role. We have argued that practiced religious faith contributes to a better quality of life.

We dare say that throughout the development of humankind, one encounters these processes constantly with recurring regularity. Social interactions shape man in a specific direction and give him what makes him different from animals—they give him will and a transcendental dimension. Social interactions lead to change and affect a person in several ways, and therefore we can study them in several contexts. First, we can talk about the personal, social, cultural and environmental context [22], which we can adapt to the issue of the relationship system. Building a relationship is very closely connected with the social interactions that the child enters at an early age. Second, the cultural context of existence can be understood as the effect of a broader social environment composed of certain norms, standards and regulations [58]. The social system, which is formed by society and its rules, is an important aspect of social relations. These are significantly limited during a pandemic [7]. Social behaviour in isolation changes significantly and is more widely understood as a product of cultural norms in interaction with biological agents. The influence of culture results in the emergence of certain regulatory norms, which are transformed into personal moral and value imperatives of man in cooperation with education and biological factors. They then regulate his behaviour. This is strongly associated with expectations in the form of the religious denomination’s behaviour and other social groups of which one is a part. 

Cultural standards relate to social interaction, as cultural patterns guide man’s cultivation and cultural norms assess the correctness of one’s actions towards others. The religious denomination becomes an important factor in believers that helps to perceive the situation positively or maintains hope throughout their lives. Differences in the degree of social interaction can be observed in different cultures. There are differences between Eastern and Western cultures, e.g., Arab women have limited social interaction. During the pandemic, we encountered restrictions in social contact, which were very strict for everyone and caused isolation. Due to the influence of social interaction in cultural communities, a social self is created, which is also conditioned by the identification and internalisation of cultural standards. The creation of the social self means, at the same time, the identification of one’s social significance and also of what we will expect from our social interactions, i.e., affirming one’s self-perception. The religious denomination is an important factor in helping during a pandemic through pastoral and psychological activities. The positive perception of these activities was more strongly perceived by people who are part of the religious denomination community. Therefore, we can state that social interactions impact the overall humanisation of man, which has been taking place over several centuries [59]. Despite this trend, our development makes us unique, authentic and autonomous people. Particular contexts of social interaction are powerful developmental components that support the relationship’s stability. Suppose no significant changes in an individual’s life in the personal, social, cultural or environmental context. In that case, we can assume that the relationship style is unchanged for him. The relationship with God as a relational person could have been significant concerning the religious denomination as a similar secular effect during pandemic isolation.

Following the implementation of pastoral and psychological service activities, we can consider in practice the use of the attachment process concept to improve people’s well-being. During the pandemic, the pastoral and psychological service started bumping into traditional ideas about religion and religious affiliation. This topic is very closely connected to the psychology of religion and attachment theory [45]. As evidenced by the research results, only people with religious affiliation belonged to the religious denomination’s activities, but they did not participate in pastoral activities despite their fear. We can only assume what caused negative emotions and reduced awareness of the community and the environment to prevail. Activities related to the religious denomination’s activities and the responsibility for pastoral and psychological service during the COVID-19 pandemic remained with the religious denomination participants themselves. The research results showed that people with religious affiliations perceived a higher level of well-being. This can also be caused by a higher ability to relate to God by building an attachment. 

The process of building a secure bonding to God as an attachment figure is connected to the topic of attachment theory. Abroad, attachment building and its existence are addressed by several influential authors [60]. The first mention of this topic—the connection between Christian theology and Bowlby’s attachment theory—was identified in 1981 by a theologian Gordon Kaufman (1925–2011), who correlated “the idea of God” to attachment figure [61]. Early research findings have shown that religious beliefs are related to differences in attachment styles [62,63]. Psychology of religion and attachment theory research indicates that belief in God can fulfil the criteria of an attachment figure. Likewise, individual differences in attachment can build correspondence or compensation pathways.

Pastoral and psychological service and attachment with God can be seen as critical factors in experiencing a positive level of well-being and quality of life [6]. The type of attachment that a person forms in childhood may be crucial during a period of isolation and inability to saturate the needs of the religious denomination’s community life during a pandemic. The attachment figure is an entity for whom the relationship becomes a model for a child. This attachment model builds further aspects of all relationships in their life. The characteristics of bonding to an attachment figure are transferred to other influential key individuals during life. With the loss of these key individuals, one has a challenging time coping [64]. Aspects of this relationship that distinguish it from other important relationships can be characterised by the closeness of the guardian in threatening situations, the care and protective function of this relationship, and the feeling of security. On the other hand, in case of separation, a feeling of anxiety arises, in the event of loss comes grief. These aspects we consider as criteria that may also determine a person’s relationship with God [65].

It is easy to draw analogies between beliefs about God and mental models of attachment figures, but it is a difficult distinction to make that God “really” can be an attachment figure. The attachment criteria of proximity are defined explicitly by the attachment figure’s availability and physical proximity. The child needs to feel the mother’s physical closeness. At an early age, the child cannot perceive the stability of the mother’s object. Proximity is how a child maintains an idea of her existence and derives her availability from it. In the case of God as an attachment figure, it is difficult to perceive this criterion, as God is not perceivable. Information about God is usually untestable empirically. However, its abstract presence in the Judeo-Christian tradition is stimulated by experiencing God’s presence through anthropomorphism and facilitation of psychological closeness by objects (e.g., icons, images, crosses, religious denominationes), or thoughts—prayer [66]. These activities are carried out mainly by the individual, even when experiencing a critical or threatening situation. Stimulating the image of God and a feeling of his presence is a frequent and emotionally powerful experience in these situations.

The protective function of the attachment behaviour, which people need to perceive during a pandemic, manifests itself, especially when the situation evokes danger, fear, or distress. These circumstances also include illness, exhaustion or separation, or the threat of separation from attachment figures [64]. Research points to the fact that people are more prone to relate to God in difficult life situations with a negative context [67]. Since people turn to pray to God and not to the religious denomination as an organisation, we can see the importance of this relationship. We can also see its importance in activating bonding and choosing coping strategies. Experiencing loss, which activates bonding behaviour, is also associated with an increased incidence of religious behaviour [65].

Beliefs about God as an attachment figure, who protects a person, are not unique. God is perceived as an entity who is omnipresent, omnipotent, and provides security. When he is lost or separated, feelings of sadness and anxiety arise. These are associated with death and eternity with or without God, with the end of faith in God, or activities related to the religious community [66]. However, research associated with the attachment to God—such as religious faith giving believers a sense of optimism and hope for the future—suggests that at least some forms of religiousness are associated with a confident, self-assured approach to life that a secure base is thought to provide [68].

If God is an attachment figure, this should pose implications for mental health consistent with the literature on worldly attachments. Being securely attached to God should be associated with desirable mental health, whereas being anxiously attached should correlate with poor mental health [69]. Indeed, a couple of studies have already found support for this proposition. The first one [60] reported that secure attachment to God was associated with greater life satisfaction and lower levels of anxiety and depression. A follow-up study [63] found that women’s secure attachment to God was inversely associated with loneliness. In the most recent study on this topic, [70] found that avoidant attachment to God, as the inverse of secure attachment, was inversely associated with symbolic immortality and agreeableness. On the opposite side, anxious attachment was positively associated with neuroticism and negative affect and inversely correlated with positive affect. 

These recent studies of attachment to God are not the only ones relevant here. Like the research linking attachment styles with mental health, literature on God imagery suggests that perceptions of the object of attachment may also pose important implications for psychological well-being [69]. Therefore, we can say that the relationship with God has very similar aspects to the relationship with a parent and thus can meet the definition of an attachment figure. Because in the context of attachment theory, we also connect the bonding to the animal, the place, close relationships, or working relationships. We can also create a parallel with the attachment to God. 

Religious-minded people, who usually belong to certain religious communities, have positive attitudes not only to ecclesiastical authorities, but also to the ability to obey certain rules (norms). This helps them to better and thus jointly accept the decision of the superior of the ecclesial community and thus to define the problems lurking from the external, i.e., secular (secularised) environment. This conservative attitude becomes, to a certain extent, a means of obedient self-discipline, which mentally strengthens (strengthens) these people, while opening up a transcendent space for them to become more fundamental inhabitants of the Czechia.

The pastoral and psychological service of the religious denomination can be connected with well-being as a positive factor during the pandemic because the social and attachment context of the religious denomination can saturate the needs of social interaction in case of social isolation. Therefore, we can say that religious affiliation creates significant skills to notice pastoral and psychological service activities. This fact can be connected with a network of religious denomination and the cooperation of religious denomination members. These people are accustomed to regular meetings, and social interaction is connected with their rules and responsibilities and community activities [71,72,73]. 

Religion is sometimes understood reductionistically [74] in the understanding of rationalism, moralism, or irrationalism. Rationalism is associated with an understanding of God and rationally knowing Him. However, personal love and respect for God are perceived as unnecessary. Contrary to this understanding, moralism focuses mainly on fulfilling obligations to the environment while rejecting its worship. Emotional living toward God highlights irrationalism but underestimates the knowledge and worship of God. This distorted concept of God can bring an unwanted dimension of religiosity.

The image of God and the relationship with God are central aspects of religiosity. These aspects are related to human behaviour, thinking, emotions and attitudes [75]. God can be an attachment figure because God provides a sense of security and safety to individuals (a “safe base” in attachment theory terms) and a “safe haven” when facing threats of potential harm. As these are two important characteristics of secure attachment in mother-infant relationships, believing one has a secure attachment to God is a particular case of believing one has a positive relationship with God. Accordingly, the studies found that believing one has a secure attachment to God has a salubrious association with mental health, including psychiatric symptoms. Conversely, believing one has an anxious or avoidant attachment to God has a pernicious association with ill mental health, including psychiatric symptoms [76,77]. 

Our study came to similar conclusions because we demonstrated the relationship between well-being and a positive experience of pastoral activity. At the same time, based on the research results, we know that people with religious affiliations have a stronger perception of the religious denomination’s activities during a pandemic and go through mainly life satisfaction. 

## 5. Conclusions

Following the results from the qualitative part of the issues related to the pandemic, we identified several topics. First, based on the answers of the research participants, we can consider that the mourners did not have many opportunities to say goodbye to the dying person or attend his funeral. Second, the pastoral activity could be perceived by society as insufficient, but it was also significantly restricted. Restrictions were tied to the religious denomination’s activities and the free movement of persons, which also caused people to consider participating in community life only in certain circumstances. Social isolation caused people to become averse to several community activities. Despite the situation, they could not even perceive the nationwide activity of the religious denomination, which was a significant step for the public. From a psychological point of view, we can talk about numbness against the supportive activity of a large part of the population, which is not only influenced by belonging to a specific religion. This may be limited by a reduction in tolerance [4] and authenticity [23] in survival during a COVID-19 pandemic crisis. The lack of social contacts and the inability to provide pastoral and psychological services could have the effect of dulling emotional experiences. The manifestation of such behaviour may be linked to inappropriate forms of attachment, which have also manifested themselves with the religious denomination and God attachment.

Our investigation has several limitations that have emerged during data collection and evaluation. Moreover, we note that the study did not test closeness or attachment to God directly, which can be proven in the following research. First, the participants in the research sample and how the researchers obtained them may be questioned because of the purposeful acquisition of the researchers.

The motivation of the research participants could be influenced by the form and duration of the testing that took place online. Therefore, the relevance of our claims may be partly related to this fact. Second, the emotional variables used have traditionally been related to each other. The methodological design was based on emotional variables and did not include psychosocial or individual variables. Third, our sample is socio-demographically heterogeneous so that the results may change in populations with different sociodemographic characteristics. Future studies should replicate these data in order to clarify the relationship between the variables studied. Finally, we recommend using our result as the basis for the subsequent research studies’ hypotheses to confirm our findings.

Our study focused on the correlations between well-being and positive experience with pastoral and psychological service during the pandemic in Czechia. Based on our results, we can draw several conclusions. First, the results confirmed the differences between well-being and positive experience of pastoral and psychological service based on religious affiliation. Furthermore, we confirmed the hypothesis of a positive correlation between well-being and positive experience with pastoral and psychological service of the religious denomination in Czechia. The results can help the performance of pastors or psychologists better understand the situation-related aspects of human experience with religious denomination as a factor of well-being [74]. Moreover, this information can be used to advise problems during the pandemic and increase motivation to realise pastoral and psychological service.

## Figures and Tables

**Table 1:** Descriptive statistics of well-being (SWLS) and experience of pastoral and psychological service (PPSE) score based on non/religious affiliation in the Czech health population.

Scale	Religiosity	N	M	SD	Min	Max
SWLS	religious	584	18.45	4.09	11	25
	nonreligious	542	14.74	4.31	6	22
PPSE	religious	584	43.50	5.94	37	50
	nonreligious	542	22.50	4.85	12	33

**Table 2:** Differences between well-being (SWLS) and experience of pastoral and psychological service (PPSE) score based on non/religious affiliation in the Czech health population.

Scale	Religiosity	N	U	Sig
SWLS	religious	584	10.537	0.015
	nonreligious	542		
PPSE	religious	584	34.031	0.000
	nonreligious	542		

## Data Availability

The data presented in this study are available on request from the corresponding author.
